# Assessment of Radiological Safety of Ceramic Tiles Commonly Used in Polish Buildings

**DOI:** 10.3390/ma18010052

**Published:** 2024-12-26

**Authors:** Aneta Łukaszek-Chmielewska, Marzena Rachwał, Joanna Rakowska, Jakub Ośko, Marta Konop, Bogdan Kosturkiewicz, Mateusz Kosturkiewicz, Marcin Łapicz

**Affiliations:** 1 Institute of Safety Engineering, Fire University, 52/54 Słowackiego St., 01-629 Warsaw, Poland; alukaszek@apoz.edu.pl (A.Ł.-C.); jrakowska@apoz.edu.pl (J.R.); mlapicz@apoz.edu.pl (M.Ł.); 2 Radiation Protection Measurements Laboratory, National Centre for Nuclear Research, 7 A. Sołtana St., 05-400 Otwock, Poland; jakub.osko@ncbj.gov.pl (J.O.); marta.konop@ncbj.gov.pl (M.K.); 3 The Faculty of Mechanical Engineering and Robotics, AGH University of Krakow, al. Mickiewicza 30, 30-059 Kraków, Poland; kostur@agh.edu.pl; 4 Jagiellonian University in Kraków, al. Mickiewicza 9A, 31-120 Kraków, Poland; mateusz.kosturkiewicz@uj.edu.pl

**Keywords:** ceramic tiles, natural radioactivity, hazard indicators

## Abstract

The concentration of natural radionuclides ^226^Ra, ^232^Th and ^40^K in ceramic tiles manufactured in Poland is presented in this paper. The concentration of natural radioactive isotopes in the tested samples was determined using a low-level digital gamma ray spectrometer equipped with an HPGe semiconductor detector. The mean concentrations of ^226^Ra, ^232^Th and ^40^K in the analyzed samples were found to be 48 ± 3 Bq∙kg^−1^, 49 ± 3 Bq∙kg^−1^ and 476 ± 23 Bq∙kg^−1^, respectively. The world mean concentrations of these radionuclides (50 Bq·kg^−1^, 50 Bq·kg^−1^ and 500 Bq·kg^−1^, respectively) were not exceeded. Furthermore, in order to ascertain the level of gamma radiation exposure, fundamental radiation protection parameters were established: radioactivity concentration indicator/gamma ray indicator (I_γ_), indoor dose rate (D_in_) and annual indoor effective dose (E_in_). In the case of the investigated ceramic tiles, it was established that the parameters were not higher than the limit values, except the indoor gamma radiation dose rate which was found to be 1.5 times higher than the world average. Therefore, the findings of this study indicate that the utilization of the examined ceramic tiles in constructions should be approached with a degree of caution.

## 1. Introduction

Poland, alongside Spain and Italy, is one of the primary producers of ceramic tiles in the European Union. The significant demand for such tiles in Poland can be attributed to various factors, including aesthetic appeal and acoustic isolation properties. Additionally, the relative affordability and thermal insulation capabilities of ceramic tiles contribute to cost-effective solutions for both winter and summer seasons. These building materials are employed in both modern construction and the restoration of existing structures [1,2].

The natural radioactive isotopes (naturally occurring radioactive materials (NORMs)) present in building materials, the Earth’s crust, air, water and food exhibit a range of concentrations. The principal radionuclides encompassed by NORMs are those that exhibit prolonged half-lives and are integral to the natural radioactive series, including uranium (^238^U), radium (^226^Ra), thorium (^232^Th) and potassium (^40^K) [3]. In light of the above, various international organizations have introduced standards that will help protect the population from the adverse effects of ionizing radiation on health. These include the International Commission on Radiological Protection (ICRP, 1994) [4], the European Commission (1999) [5] and United Nations Scientific Committee on the Effects of Atomic Radiation (UNSCEAR, 2000, 2008) [6,7]; the European Atomic Energy Community (EAEC, also Euratom) [8] and the Council of the European Union [9] introduced radiological protection standards with the objective of safeguarding the population from the adverse effects of ionizing radiation on their health. The mean global concentrations of the naturally occurring radioactive isotopes ^226^Ra, ^232^Th and ^40^K in building materials are 50, 50 and 500 Bq·kg^−1^, respectively [10,11,12,13,14]. The radioactivity of ceramic tiles is contingent upon the concentration of natural radioactive isotopes present in the raw materials employed in their production. The initial raw materials employed in the production of ceramic tiles are clay, kaolin, feldspars and natural mineral and synthetic dyes, which are formed as a consequence of the chemical weathering of igneous, metamorphic or sedimentary rocks. The concentration of natural radioactive elements present in ceramic tiles is dependent upon the type of rock from which they are derived, their geological origin and the depth at which they are extracted [15].

In light of the findings regarding the concentrations of natural radioactive isotopes in raw materials (kaolin, feldspar, quartz, zircon, clay, dolomite) utilized in ceramic tile production (Table 1), it is evident that zirconium exhibits the highest natural radioactivity among the raw materials. Zirconium pigment is a synthetic inorganic product with the ability to color ceramic glasses yellow-green.

Zirconium is used in small quantities and its significant contribution to the increase in the radioactivity of ceramic tiles is limited to the coloring of tiles. The primary raw materials exhibiting elevated radium and potassium concentrations are feldspars and clay [23]. As can be seen, each raw material has specific concentrations of radionuclides, which are the source of the radiation in the ceramic tiles. Despite the existence of studies on the content of radionuclides in ceramic tiles in the world literature, no such studies have been carried out in Poland. For many years, ceramic tiles have been a ubiquitous product, commonly purchased and utilized in residential properties, public utility buildings, and so on. It can be posited that every individual has contact with them on a daily basis. Despite the numerous restrictions on radioactivity in the human environment, building materials, particularly in Poland, frequently fail to meet the standard control parameters for radionuclide content. This can result in their accumulation in the human environment, thereby posing a significant health risk. Consequently, the authors of the present study aimed to examine and assess the most commonly used building materials in this respect, with ceramic tiles being a primary focus. An assessment was conducted on the basis of the concentrations of naturally occurring radioactive isotopes (^226^Ra, ^232^Th and ^40^K) in ceramic tiles produced in Poland and employed in construction. Based on the determined concentrations of natural radioactive isotopes, the gamma radiation index (I_γ_) was calculated. In Poland, I_γ_ is referred to as the radioactive concentration index and is the sole determining factor in the suitability of a given material for use in construction. Furthermore, the indoor dose rate from ceramic tiles, as well as the annual effective radiation dose rate, was calculated.

## 2. Materials and Methods

### 2.1. Sampling and Sample Preparation

**Figure 1:**
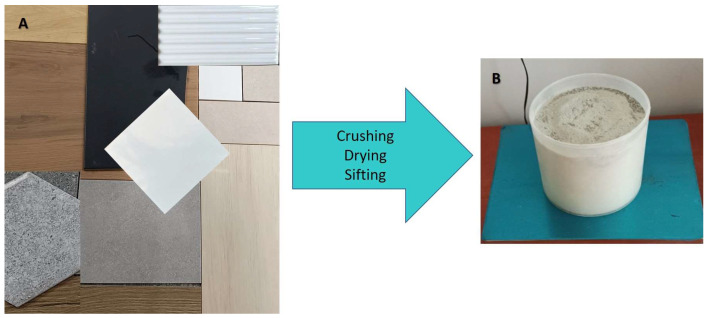
The ceramic tiles purchased for use in the research (**A**) and pre-prepared sample in the Marinelli vessel (**B**).

Poland-produced ceramic tiles were purchased in DIY stores (Figure 1). The fundamental raw materials employed in the manufacture of ceramic tiles are clay, kaolin, quartz and feldspar, which are combined in the optimal proportions to create the ideal mixture for tile production. The accurate selection of materials and their proportions, crucial for the quality of the final ceramic tiles, remain a closely guarded secret of the tile manufacturers.

Poland is a country relatively rich in kaolin mineral deposits (the resources of 16 deposits in 2023 amounted to 225.53 million tons [32]). Almost all kaolin mineral extraction in Poland comes from the Maria III kaolinite sandstone deposit in Lower Silesia. Additionally, mud kaolin is recovered from sands and quartz sandstones of the Bolesławiec and Tomaszów Troughs as a by-product of their washing. On the other hand, the basic sources of feldspar (feldspar–quartz) raw materials are granitoid deposits located in the Strzegom-Sobótka massif in Lower Silesia. Ceramic tiles are also made of fractions rich in alkalis, which are created during the current production of crushed aggregates in Lower Silesian granite mines, including Graniczna, Rogoźnica and Gniewków. The domestic resource base of feldspar minerals is small and covers mainly the area of the Izera Foothills and the Strzegom-Sobótka granite massif. Feldspar–quartz raw materials of domestic origin are characterized by poor quality, which makes it necessary to import them from other countries. The main suppliers of feldspar–quartz raw materials to Poland are Turkey and the Czech Republic, while Norway is the supplier of nepheline syenite [33].

Tiles samples were crumbled and dried at a temperature of 105 °C for 24 h to constant weight. Afterwards, they were sifted through sieves with 2 mm diameter mesh during which the samples were compacted at 50 Hz frequency with a vortex shaker. The samples were then placed in 0.645 dm^3^ Marinelli vessels in order to achieve geometrically uniform readings. Each sample was subsequently weighed and then set aside for 30 days to obtain a radioactivity balance between ^226^Ra and ^214^Bi; ^232^Th and ^208^Tl; as well as ^226^Ra and ^222^Rn. Thirty days is enough time to achieve secular equilibrium—a situation when the quantity of a radioactive isotope remains constant because its production rate (e.g., due to decay of a parent isotope) is equal to its decay rate. The weight of a single sample was within the range of 0.67–1.01 kg.

### 2.2. Measurements

**Figure 2:**
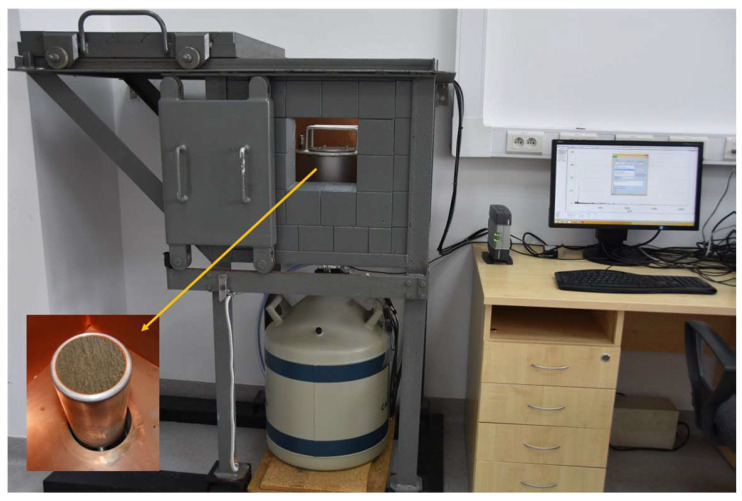
Picture of the HPGe spectrometric set with a view of the sample placed in the sample chamber.

**Figure 3:**
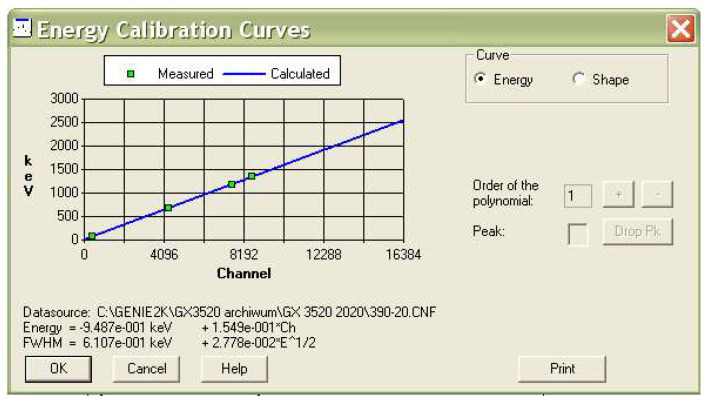
The calibration curve: energy (keV) versus channel number.

The concentrations of natural radioactive isotopes in the samples were determined using a low-background spectrometer (Figure 2) with an HPGe semiconductor detector (with a detection efficiency of 35% and energy resolution of 1.73% at 1332 keV for the analyzed geometry). The detector was calibrated using Laboratory SOurceless Calibration Software (S574 LabSOCS Calibration Software, version 4.2) provided by MIRION Technologies, taking into account the sample material and density. LabSOCS uses the Monte Carlo method to create a mathematical calibration curve of the performance of semiconductor detectors (Figure 3). This is performed without the use of radioactive sources when measurements are taken in a laboratory. This method is most often used when it is impossible to provide a measurement geometry that would fully correspond to the measured samples [34]. The Genie 2000 Spectroscopy Software was used for spectra visualization and all calculations. The semiconductor detector allowed the measurement of even the low-efficiency radioactive decay isotopes. ^226^Ra radioactivity was determined according to the ^214^Bi line (609 keV), assuming radioactive equilibrium between these isotopes. The ^232^Th isotope was determined based on its decay products, namely ^228^Ac (338.40, 911.07 and 968.90 keV). The activity of ^40^K was determined directly from its gamma emission at 1460.83 keV. The measurement time for each sample was 64,800 s. This is enough time to achieve an appropriate statistical uncertainty of the number of counts for each of the energy peaks.

### 2.3. Calculations and Statistics

For each result, basic statistical parameters were calculated: mean, standard deviation, maximal and minimal radiological parameter value. Additionally, Pearson correlation coefficients between indicators (I_γ_, D_in_, E_in_) and concentrations of natural radionuclides (^226^Ra, ^232^Th and ^40^K) were determined (assuming a significance level *p* below 0.05).

Poland, just like every other EU member state, is bound to abide by harmonized regulations in regard to construction products’ quality when entering the market. The primary Polish act stipulating these requirements is Ordinance of the Council of Ministers dated 17 December 2020 [35] in regard to construction materials and the concentration of their radionuclides (^226^Ra, ^232^Th and ^40^K), labeling such materials and informing competent authorities about exceeding radionuclide concentration norms. This act stipulates the criteria that have to be met in order to use construction materials. One of them is the radioactivity concentration indicator I_γ_, which determines the exposure of the human body to gamma radiation emitted by radium, thorium and potassium that are present in building materials. The value of the I_γ_ index is calculated with Equation (1) [5,35]:

(1) $$I_{\gamma }=\frac{A_{Ra}}{300}+\frac{A_{Th}}{200}+\frac{A_{K}}{3000}$$

where:

I_γ_—gamma radiation indicator/radioactivity concentration indicator (-);
A_Ra_—radium specific activity (Bq∙kg^−1^);
A_Th_—thorium specific activity (Bq∙kg^−1^);
A_K_—potassium specific activity (Bq∙kg^−1^).

Total measurement uncertainty of the I_γ_ indicator cannot exceed 20% of its value if it is not lower than 0.8. If the value is higher than 1, then there is a hazard of exceeding the reference value that is dangerous to humans due to high gamma radiation emitted by the construction materials which equals 1 mSv annually. In this case, construction supervision authorities have to be consulted [35].

Additionally, the dose rate of indoor gamma radiation [nGy∙h^−1^], as well as an annual indoor effective dose E_in_ [mSv∙y^−1^] emitted by ceramic tiles—Equation (2) [5] and Equation (3) [7]—were calculated. Similarly to Equation (1), A_Ra_, A_Th_, and A_K_ indicate radium, thorium and potassium concentration in Bq∙kg^−1^ in the samples.

(2) $$D_{in}=0.92A_{Ra}+1.1A_{Th}+0.080A_{K}$$

(3) $$E_{in}=D_{in}\cdot 8766\cdot 0.8\cdot 0.7\cdot 10^{-6}0.080A_{K}$$

## 3. Results and Discussion

### 3.1. Radionuclide Concentration in the Ceramic Tile Samples

**Figure 4:**
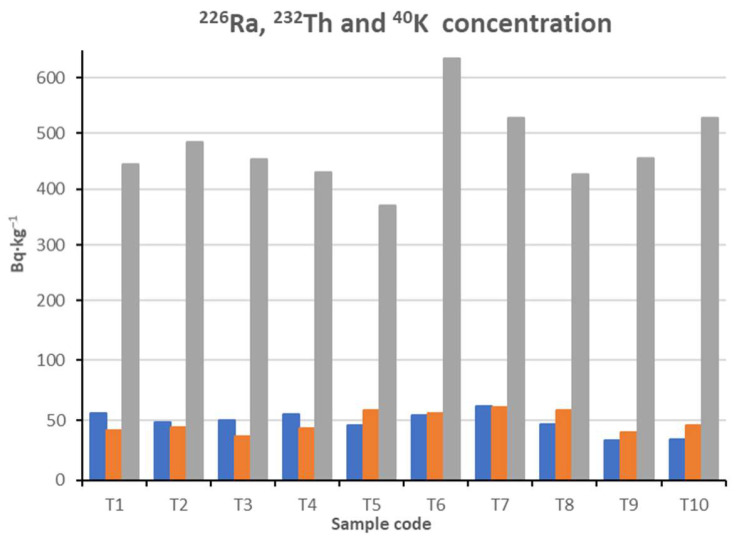
Concentrations of ^226^Ra—■, ^232^Th—■ and ^40^K—■ in examined ceramic tile samples.

The concentration levels of radium, thorium and potassium radionuclides in the ceramic tile samples produced in Poland exhibit variability (Table 2, Figure 4). The primary isotope responsible for the natural radioactivity of these tiles is ^40^K, followed by ^232^Th and ^226^Ra.

Mean concentration of radionuclides ^226^Ra, ^232^Th and ^40^K amounted to 48 ± 3 Bq∙kg^−1^, 49 ± 3 Bq∙kg^−1^ and 476 ± 23 Bq∙kg^−1^, respectively. Sample T7 contained the highest concentration level of radium and thorium whereas sample T6 contained the highest concentration level of potassium. In comparison with the world mean (the concentration of aforementioned isotopes [10,11,12,13]), sample T7 contained higher concentrations of all three isotopes. Samples T1 and T4 contained slightly higher levels of radium in relation to the world mean. Similarly, samples T5, T6 and T7 contained marginally greater concentration levels of thorium, whereas T6 and T10 contained higher levels of potassium.

The aforementioned results were compared with the findings of other scientists’ research, and the mean concentration of the ^226^Ra isotope in domestic material was found to be comparable with the mean of ceramic tiles manufactured in various locations (Table 3), including Egypt (Lecico and El-Gawhara), Algeria (T13), Italy (T16) and Greece (T22). The highest concentration of radium was observed in ceramic tiles produced in Egypt (samples T12, T14 and T23), China (sample T15), Palestine (sample T18), Yemen (sample T19) and Nigeria (sample T20). Conversely, the lowest concentrations of radium were found in Cameroon (sample T17) and India (sample T24).

Subsequently, the mean concentration of the ^232^Th isotope in domestic material is comparable with the mean in ceramic tiles manufactured in Algeria—T13, Italy—T16 and Greece—T22. Higher concentrations of thorium in relation to Polish tiles were found in samples from Egypt—T12, T14 and T23, China—T15 and T25, Palestine—T18, Yemen—T19, Nigeria—T20, India—T24, Turkey—T26 and Serbia—T27. Lower concentrations of radium were found in samples T11 and T17 (Table 3).

Finally, mean concentration of the ^40^K isotope in domestic material is comparable with the mean in ceramic tiles manufactured in Egypt—T11, Italy—T16, Nigeria—T20, China—T25 and Turkey—T26. Higher concentrations of thorium in relation to Polish tiles were found in samples from Egypt—T14, Palestine—T18 and Serbia—T27. Lower concentrations of radium were found in samples from Egypt—T12 and T23, Algeria—T13, China—T15, Cameroon—T17, Yemen—T19, Greece—T22 and India—T24 (Table 3).

Considering that the radium content in construction material averages 50 Bq∙kg^−1^, an increased level of this radionuclide was identified in samples from Egypt (T12, T14 and T23), Palestine (T18), Yemen (T19), Nigeria (T20), China (T25), Turkey (T26) and Serbia (T27). A comparison of the ^232^Th isotope concentration in the tiles with the global mean in building materials reveals an increase in the samples from Egypt (T12, T14 and T23), China (T15), Yemen (T19) and Nigeria (T20). With regard to potassium, an elevated level of this isotope was observed in the samples from Egypt (T14), Palestine (T18) and Serbia (T27).

A comparison of the findings regarding Polish ceramic tiles with the results of other scientists’ research indicates that the natural isotope concentration in ceramic tile distribution is not uniform. Consequently, further research in this field is recommended. The majority of construction materials referenced in this paper exhibit elevated concentrations of ^226^Ra, ^232^Th, and ^40^K radionuclides in comparison to global averages. In contrast, Polish construction materials display comparable or even lower levels of these concentrations relative to global averages.

### 3.2. Radioactivity Index Value

Table 4 presents the radioactivity index value, the dose rate of indoor gamma radiation and annual effective dose for the sampled ceramic tiles manufactured in Poland.

Sample T9 scored the lowest value of the indicator I_γ_ = 0.46 ± 0.02; sample T7 scored the highest for the same indicator I_γ_ = 0.69 ± 0.01; whereas the mean value for all of the samples equalled 0.56 ± 0.02. None of the analyzed samples exceeded the limit value of the radioactivity concentration level indicator. Hence, the analyzed ceramic tiles can be used in construction industry both in Poland and any other member state of the EU.

The dose rate of indoor gamma radiation of the examined materials varies within the range of 110–166 nGy·h^−1^. The highest dose rate was observed in Sample T7, while the lowest was recorded in T9. The mean dose rate for all samples was found to be 126 ± 5 nGy∙h^−1^. This value is higher than the mean world gamma radiation dose rate indoors, which is 84 nGy∙h^−1^. Therefore, it can be concluded that the use of ceramic tiles indoors contributes to an increase in the aforementioned gamma radiation dose rate.

The annual indoor effective dose value of ceramic materials ranges within the range of 0.54–0.82 mSv·y^−1^. The lowest E_in_ value was observed in T9, while T7 exhibited the highest value. The mean annual indoor effective dose rate was found to be 0.67 ± 0.03 mSv·y^−1^. In light of the estimated annual indoor effective dose rate of 0.41 mSv∙y^−1^, it can be posited that the utilization of ceramic tiles indoors is a contributing factor to an increase in E_in_. The calculated annual indoor effective dose (E_in_) for the tested ceramic samples is considerably below the ICRP-60 [4] criterion limit of 1 mSv∙y^−1^.

The data presented above indicate that the use of ceramic tiles indoors may result in an increase in the dose rate of gamma radiation. However, the primary consideration in the approval of construction materials is not the dose rate but the radiation concentration level. Consequently, all of the examined tiles can be used in the construction industry in Poland and the EU.

The correlation coefficient, a statistical metric used to quantify the strength of a linear relationship between two variables, was calculated. This analysis revealed a strong relationship between the radioactivity concentration indicator and the thorium concentration, as well as a moderate relationship between the radium concentration and the radioactivity concentration indicator. Furthermore, a pronounced relationship was observed between the dose rate of indoor gamma radiation and the annual indoor effective dose. Correlation between the concentrations of thorium and radium was conformed. However, the correlation coefficient for potassium was found to be statistically insignificant in this instance, as evidenced by a *p*-value greater than 0.05 (Table 5).

## 4. Conclusions

In this paper, the radioactivity concentration of the natural radionuclides ^226^Ra, ^232^Th and ^40^K in ceramic tiles was determined by low-level gamma spectrometry. The results of the natural radionuclide concentration enabled the authors to ascertain the fundamental principles of radiation protection. The mean concentrations of ^226^Ra, ^232^Th and ^40^K in the analyzed ceramic tiles were found to be 48 Bq·kg^−1^, 49 Bq·kg^−1^ and 476 Bq·kg^−1^, respectively. Given that the world mean concentrations of the aforementioned isotopes are 50 Bq·kg^−1^, 50 Bq·kg^−1^ and 500 Bq·kg^−1^, respectively, it can be posited that tiles manufactured in Poland contain comparable levels of those isotopes. It should be noted, however, that their concentrations exceed the values recommended by the European Union [5] (40, 40 and 400 Bq/kg corresponding to ^226^Ra, ^232^Th and ^40^K). It is also noteworthy that ceramic tiles are not the only product used in interior trims. Other materials, such as granite floors and windowsills, tiling adhesives, ceramics filler, plaster or parget, can also contribute to the annual indoor effective dose being exceeded. It is therefore imperative to monitor the concentration of natural radionuclides in construction materials in order to avoid exposing people to additional radiation hazards.

Furthermore, substantial radiation protection parameters were established, namely the radioactivity concentration indicator (I_γ_), the indoor gamma dose rate (D_in_) and the annual indoor effective dose (E_in_). The mean value of I_γ_ did not exceed 1, indicating that the examined ceramic tiles can be used in the construction industry. The indoor gamma radiation dose rate was found to be 1.5 times higher than the world average of 84 nGy·h^−1^, indicating an elevated concentration of natural isotopes in the analyzed materials. Furthermore, when considering the calculation method of this index (see Equation (2)), it is evident that an increased concentration of radium and thorium, exceeding the EU recommended value of 40 Bq kg^−1^, exerts an influence on the calculated value of D_in_.

## Figures and Tables

**Table 1:** Concentration of radionuclides ^226^Ra, ^232^Th, ^40^K in raw materials used in ceramic tile manufacturing.

Raw Material	Country of Origin	Concentration [Bq∙kg^−1^] *			Reference
		^226^Ra	^232^Th	^40^K	
Kaolin	Nigeria	18.5–64.2 (38.17)	48.7–98.3 (65.11)	31.3–156.5 (93.90)	[16]
	Turkey	16.5–125.9 (82 ± 37)	24.0–184.0 (95 ± 49)	15.0–1635.2 (464 ± 545)	[17]
	Egypt	59.8–8499	95.8–1079	8.2–269.7	[18]
	Serbia	178 ± 15	87 ± 12	400 ± 80	[19]
	Czechia	77 ± 9	163 ± 21	220 ± 70	[20]
	Bosnia and Herzegovina	124 ± 9	109 ± 8	1170 ± 80	[20]
	India	13.6–113.5 (51.4)	30.5–169.4 (90.6)	54.7–338.9 (201.4)	[21]
	East Asia	8633 ± 432	1079 ± 54	134.3 ± 6.7	[22]
	Poland	45.3 ± 0.9	46.8 ± 0.1	175.2 ± 3.5	[23]
Feldspar	Poland I	65.8 ± 1.2	12.6 ± 0.5	904.5 ± 8.3	[23]
	Poland II	379.3 ± 3.0	12.6 ± 0.5	917.8 ± 8.4	[23]
	Germany	28 ± 5	16 ± 5	3620 ± 270	[20]
	Czechia	16 ± 2	11 ± 2	3580 ± 130	[20]
	Ukraine	10.4 ± 2.5	10.4 ± 2.6	3810 ± 220	[20]
	Serbia	<5–158 (54 ± 29)	<3–163 (69 ± 40)	<10–3192 (320 ± 760)	[22]
	India	5.7–40.2	4.8–51.6	24.7–2113.2	[21]
	Egypt	9.5–183675.7	6.1–94314.2	0–7894.6	[24]
Quartz	Poland	8.2 ± 0.4	5.9 ± 0.3	7.6 ± 0.4	[23]
	India	6.7–38.3 (24.1)	9.4–43.7 (28.4)	50.3–302.4 (189.1)	[21]
	Serbia	<5–94 (21 ± 24)	<3–385 (61 ± 120)	<10–340 (46 ± 95)	[25]
	Russia	282 ± 15	382 ± 17	150 ± 15	[20]
	Turkey	3.1–43.4 (14 ± 3)	2.9–44.8 (11 ± 3)	49.5–485.8 (222 ± 48)	[26]
Zircon	India	1324.2–1874.3 (1592.9)	197.2–346.5 (273.4)	64.3–194.7 (118.7)	[21]
	Italy	1690–6900 (3381 ± 209)	180–1050 (483 ± 40)	25–700 (204 ± 20)	[27]
	Serbia	4746–4938 (4840 ± 40)	673–763 (718 ± 64)	<10–30	[24]
	Spain	1570–4920 (3161 ± 229)	145–700 (388 ± 27)	30–280 (173 ± 21)	[27]
	Germany	4000 ± 130	700 ± 80	250 ± 15	[20]
	The Netherlands	2120 ± 40	264 ± 14	150 ± 8	[20]
	Czechia	2420 ± 80	300 ± 30	190 ± 10	[20]
	Egypt	3341–3817 (3588 ± 125)	884–1063 (982 ± 47.7)	163–353 (217 ± 48)	[22]
Clay	Poland I	60.8 ± 1	80.4 ± 1.2	555.3 ± 6.5	[23]
	Poland II	47.4 ± 1	72.6 ± 1.2	504.3 ± 6.2	[23]
	Saudi Arabia	151 ± 13	170 ± 14	1144 ± 65	[28]
	Egypt	91	102	707	[29]
	Estonia	69.3 ± 3.8	82 ± 3.5	678 ± 15	[30]
	Finland	53.6 ± 3.4	58.5 ± 3.2	748 ± 17	[30]
	Germany	35.6 ± 2.3	55.2 ± 2.4	447 ± 11	[30]
Dolomite	Lithuania	34.8 ± 3.4	51.6 ± 3.1	1074 ± 34	[31]
	Poland	32.6 ± 0.9	1.7 ± 0.2	24.8 ± 1	[23]

* Indicates average concentration of natural radionuclides in the samples.

**Table 2:** Mean concentration values and standard deviation of natural radionuclides ^226^Ra, ^232^Th and ^40^K in examined ceramic tile samples.

Raw Material	Sample Code	Concentration [Bq∙kg^−1^]		
		^226^Ra	^232^Th	^40^K
Sample 1	T1	56 ± 4	42 ± 2	445 ± 28
Sample 2	T2	48 ± 3	44 ± 2	484 ± 30
Sample 3	T3	50 ± 4	37 ± 2	453 ± 29
Sample 4	T4	55 ± 4	43 ± 2	431 ± 28
Sample 5	T5	46 ± 3	58 ± 2	370 ± 26
Sample 6	T6	54 ± 4	56 ± 3	635 ± 40
Sample 7	T7	62 ± 2	61 ± 2	528 ± 9
Sample 8	T8	47 ± 2	58 ± 2	427 ± 8
Sample 9	T9	33 ± 3	40 ± 2	456 ± 30
Sample 10	T10	34 ± 2	46 ± 2	528 ± 34
Min		33	37	370
Max		62	61	635
Mean ± standard deviation		48 ± 3	49 ± 3	476 ± 23
Recommended value [5]		40	40	400

**Table 3:** Concentration of natural radionuclides ^226^Ra, ^232^Th and ^40^K in ceramic tiles produced in various countries.

Sample Number	Sample Code	Country of Origin	Concentration [Bq∙kg^−1^]			Reference
			^226^Ra	^232^Th	^40^K	
11	T11	Egypt (Lecico and El-Gawhara)	41.7–60.7 (52.2)	30.7–47.1 (39.1)	195–680 (480)	[36,37]
12	T12	Egypt (Qena)	40–230 (126)	10–130 (72)	80–600 (300)	[37,38]
13	T13	Algeria	55	41	410	[37,39]
14	T14	Egypt (Cleopatra Factory)	71.2–86 (76.1)	63.3–68.7 (66.2)	900–1018 (962)	[37,40]
15	T15	China	63.5–131.4	55.4–106.5	63.5–131.4	[12,37]
16	T16	Italy	56	43	440	[11,37]
17	T17	Cameroon	11.3–13.13 (12)	18.63–22.64 (20)	319	[37,41]
18	T18	Palestine	45.4–102.0 (73.7)	38.8–78.3 (58.2)	363–871.2 (624)	[37,42]
19	T19	Yemen	0–549 (131.88)	13–267 (83.55)	24–869 (400.7)	[37]
20	T20	Nigeria	37.5–241.0 (61.1)	41.5–126.5 (70.2)	270.0–940.0 (514.7)	[43]
21	T21	Italy	36–87	38–86	411–996	[31]
22	T22	Greece	58	46	409	[31]
23	T23	Egypt	126	72	300	[31]
24	T24	India	28	64	24	[31]
25	T25	China	73	62	480	[31]
26	T26	Turkey	70	62	477	[31]
27	T27	Serbia	72–122	59–79	723–1013	[31]

**Table 4:** Radioactivity concentration indicator value (I_γ_), the dose rate of indoor gamma radiation (D_in_) and annual indoor effective dose (E_in_) for the sampled ceramic tiles.

Sample Code	I_γ_ [-]	D_in_ [nGy∙h^−1^]	E_in_ [mSv∙y^−1^]
T1	0.55 ± 0.02	134 ± 5	0.66 ± 0.15
T2	0.54 ± 0.02	131 ± 5	0.64 ± 0.15
T3	0.50 ± 0.02	123 ± 4	0.60 ± 0.15
T4	0.54 ± 0.02	132 ± 5	0.65 ± 0.16
T5	0.57 ± 0.02	135 ± 4	0.66 ± 0.14
T6	0.67 ± 0.02	162 ± 6	0.80 ± 0.17
T7	0.69 ± 0.01	166 ± 2	0.82 ± 0.11
T8	0.59 ± 0.01	141 ± 2	0.69 ± 0.11
T9	0.46 ± 0.02	110 ± 4	0.54 ± 0.14
T10	0.52 ± 0.02	124 ± 4	0.61 ± 0.15
Min	0.46	110	0.54
Max	0.69	166	0.82
Mean ± standard deviation	0.56 ± 0.02	126 ± 5	0.67 ± 0.03
World mean	<1	84	0.41

**Table 5:** Pearson correlation coefficients (r) between radioactivity indices I_γ_, D_in_, E_in_ and natural radionuclide concentrations in analyzed samples (significant at *p* < 0.05).

Parameter	^226^Ra	^232^Th	^40^K
I_γ_	0.694 *p* = 0.026	0.8386 *p* = 0.003	0.511 *p* = 0.131
D_in_	0.744 *p* = 0.014	0.787 *p* = 0.007	0.527 *p* = 0.117
E_in_	0.742 *p* = 0.014	0.780 *p* = 0.008	0.539 *p* = 0.121

## Data Availability

The data presented in this study are available on request from the corresponding author due to privacy.
